# Personality, Intervention and Psychological Treatment: Untangling and Explaining New Horizons and Perspectives

**DOI:** 10.3390/bs12080245

**Published:** 2022-07-22

**Authors:** Casandra I. Montoro, Carmen M. Galvez-Sánchez

**Affiliations:** Department of Psychology, University of Jaén, 23071 Jaén, Spain

Personality—as a construct—is involved in both health and disease. Regardless of whether it is normal or pathological, it is considered one of the most relevant aspects in clinical and behavioral psychology. Personality and related factors (i.e., negative affect, resilience, alexithymia, perceived stress, emotional regulation, empathy, emotional dependence, locus of control, etc.) have been associated with a variety of disorders (e.g., chronic conditions such as chronic pain and cancer), psychological negative states (i.e., anxiety, depression), and even violent and criminal behaviors, among many others. Moreover, the COVID-19 pandemic and the attendant uncertainty, discomfort, and unpredictability have highlighted anew the relevance of personality in the disease process, coping, and attitudes toward therapies and health in general. Unfortunately, although, in the course of vital epochs, the coping significance of personality is frequently spotlighted, more research maintained over time and constantly updated is still crucial to reaching the goal of a personalized health approach. Therefore, better understanding the protection and risk factors associated with health and disease, wherein personality plays a relevant role, is undoubtedly essential to: (1) assure a better psychological adjustment, and (2) provide individual prevention and intervention strategies, aimed at dampening the psychological impact in people prone to suffer any kind of mental health impairment or subjected to the high demands emerging in the current challenging contexts.

This Special Issue tackles the personality research and comprehension in a collection of eight research articles authored by experts in the study of personality and/or in the specific conditions evaluated. These studies address theoretical and practical advances on personality in different populations and age groups, as well as the research findings of interventions and treatment strategies involving personality. Of the seven original research articles and one scoping review on personality included in this Special Issue, three are focused on healthy adult populations [1,2,3], two in healthy children and adolescent [4,5], other two in chronic pain conditions (migraine [6] and fibromyalgia [7]) and one in personality disorders (cluster B and C [8]). The variety of conditions, populations, personality traits and research methods used in the included studies makes difficult a direct comparison of the findings but provide a wider approach to personality, intervention, and psychological treatment. Additionally, the further benefit of this variety is that it invites discussion and continuous contribution and provides insights on behavior and personality comprehension. The Special Issue’s insights might help to improve and evaluate the current prevention and therapy approaches. Furthermore, based on the findings research gaps also arise and point out new fields of research. Following, these studies are presented and discussed in order of publication date in the special.

First, the study by Pottof et al. [1] aimed at examining—for the first time—the influence of personality in the self-viewing on a mirror and others viewing on a video using different gaze parameters (duration and fixation) and the eye-tracking technique. Gender differences in gaze parameters were also analyzed. Authors reviewed literature on self-viewing and did not find firm conclusions on what kind of feeling self-viewing in a mirror or picture provokes (e.g., dissatisfaction or enjoyment). The role of personality factors (e.g., self-esteem, self-disgust, and narcissism) was suggested to be of special relevance. Accordingly, with the abovementioned, in this research it was observed a significant negative association between self-esteem and gaze duration. In fact, people characterized by higher self-esteem looked shorter time at both own-face and other-face. Males compared to females tended to look longer at their faces. The longer gaze duration was proposed as an indicator of a higher critical evaluation of the face. Duration and fixation of the gaze were not related either narcissism or self-disgust, ruling out the role of these personality factors on self-viewing. Pottof et al. [1] concluded that self-esteem protects against critical evaluation of the faces. Unlike previous evidence supporting females as more critical of their physical appearance than males, in this study males needed more time to evaluate themselves and others (likely critically) than females. The appropriateness of including the gender perspective in personality and psychological research, with the purpose of deepening into the studied phenomena and contributing to a more tailored approach, is inferred.

The research conducted by Rodríguez-Árbol et al. [2] explored changes in the psychological wellbeing (anxiety, depression, and self-perceived health) of young healthy university students during the initial 14 days of the COVID-19 general lockdown and the role of personality (neuroticism, psychoticism, and extraversion) in the evaluated aforementioned changes. Notwithstanding several studies have been conducted in relation to the COVID-19 effects, this research is considered one of the few investigating the COVID-19 effects during the lockdown in people without previous mental conditions. The study showed a significant increase of anxiety and depression symptoms (the latter even to reach levels for mild-depression), and a decrease on self-perceived mental health and vitality throughout the lockdown. A special emphasis was directed to the observed more acute detriment in anxiety and depression during the first week followed by a plateau (stabilization) while self-perceived health kept lessening. Neuroticism, intolerance of uncertainty, and negative autofocus were related to worse levels of psychological adjustment. This study raises awareness of the neediness to include personality in the prevention and treatment programs, not only in ill populations but also in healthy ones.

The study of Romeo et al. [7] is characterized by its novelty in exploring the contribution of both defense mechanisms and personality characteristics on the psychological distress of fibromyalgia patients. Associations between personality traits (alexithymia, harm avoidance, cooperativeness, persistence), defense mechanisms (adaptive and maladaptive defense style, image-distorting, and self-sacrificing) and psychological distress (anxiety and depression) in fibromyalgia patients and healthy controls were explored. Alexithymia, harm avoidance, and maladaptive defense style were significant predictors of patients’ psychological distress. Despite the higher harm avoidance levels in the fibromyalgia patients’ group compared to the healthy control group, harm avoidance was also predictor of healthy control’s psychological distress. Surprisingly, in healthy controls, an adaptative defense style further predicted psychological distress. This last finding questions previous literature highlighting the protective and positive role of adaptive defense styles in the greater psychological wellbeing of the general population and suggests not to overlook the importance of a personalized approach in psychological evaluation. Moreover, and congruent with the latter, the need for a total avoidance of any influence in the therapist’s focus from the consideration of certain personality traits as desirable (non-pathologic) or not desirable (pathologic) is underlined. This Special Issue also contains a research study supporting this last consideration (i.e., Montoro et al. [3]).

The scoping review of Galvez-Sánchez et al. [6] had the objective of exploring in depth the relationship between migraine and neuroticism, with the purpose to provide a better treatment for migraine patients based on a personalized and more comprehensive approach. Broadly, it was confirmed the higher level of neuroticism and vulnerability to negative affect in migraine patients, compared to non-migraineurs and tension-type headache patients, and demonstrated a well-stablished association between neuroticism and migraine. Furthermore, some research gaps arose from this review. For instance, some remaining questions were if neuroticism may be a predictor of the chronification of migraine and if there exist any potential moderators that explain the relationship between neuroticism and migraine. In fact, scientists should be cautious in the analysis and interpretation of linear associations between personality factors and other psychological variables. Mediational and moderation models might be useful to better understand the role of all involved variables.

The study of Grummitt et al. [4] is a longitudinal study that investigated associations between personality (hopelessness, anxiety sensitivity, impulsivity, and sensation seeking) at age 13 and the number of traumatic events experienced by age 18. This research entails a special social and clinical relevance because the need to prevent the accumulation of traumatic events is an urgent public health priority. The main findings of this study pertain to: (1) the relation observed between high scores on hopelessness, impulsivity, and sensation seeking at age 13 and greater number of traumatic events by age 18; (2) the prediction of the number of new traumatic events from age 13 to 18 by impulsivity and the sensation seeking; and (3) the association between prior trauma exposure and high hopelessness at age 13. Findings clearly proved the presence of a high risk of experiencing traumatic events under certain personality traits (hopelessness, impulsivity, and sensation seeking), as well as the influence of these experiences in the later personality development.

With regard to the study of Montoro et al. [3], note that it was cited above as a support of an individual evaluation of personality and consequently a personalized approach to the prevention of mental health problems before a protective or risk role is inflexible assigned to certain personality traits. Without detriment to the last, it is important remark that this study was focused on the mediational role of different emotional factors (resilience capacity, emotional regulation, positive and negative affect, intolerance of uncertainty, and perceived stress) in the relationship between narcissism and post-traumatic symptoms in healthy adults. Interestingly, and in opposition to the proposed link between vulnerability to trauma and narcissistic personality traits, narcissism personality was mostly associated (with the exception of exploitativeness and entitlement dimensions) with lower post-traumatic symptoms and higher emotional adaptative outcomes. The associations between post-traumatic symptoms and narcissism were mediated by affect and resilience. These findings suggest that narcissistic traits might be, in some cases, adequate for coping with post-traumatic symptoms, supporting the notion to personalized and patient centered approaches are vital in all clinical fields (prevention, diagnosis, and treatment).

The penultimate paper of this Special Issue, by Massaal-van der Ree et al. [8], opened a path of research on cluster C personality disorders. Cluster C personality disorders are thought to be more prevalent than cluster B personality disorders, but less studied because they not represent a severe form of personality disorder. In order to test this assumption, authors compared patients with cluster B and C personality disorders on a wide range of clinically-relevant severity measures (comorbidity, suicidality, childhood traumatization, and global functioning). Cluster B personality disorders patients were prone, in accordance to the literature, to suffer from substance use disorders and showed greater suicide attempts as well as worse global functioning (fewer friendships) compared to cluster C personality disorders patients. Nonetheless, in the rest of the clinical measures (trauma variables, work-related, emotional, and social functioning), no differences were observed between cluster B and cluster C personality disorders patients. Furthermore, patients diagnosed by the combination of both cluster B and cluster C personality disorders suffered more often from comorbid anxiety disorders than cluster B personality disorders patients and had fewer friendships than patients with solely cluster C personality disorders had. Findings underscore the need for routine assessment evidence-based treatments for cluster C personality disorders patients. As informed by authors, up-to-date cluster B personality disorders patients have dominated the research agendas and treatments have been only proven in cluster B personality disorders patients. However, both of them do not significant differ on traumatization, revealing the need for more research on and evidence-based treatments of cluster B personality disorders. This might avoid the over and/or under-diagnosis of some personality patterns and in consequence an inappropriate treatment process.

Finally, the study of Torres-Fernández et al. [5] evaluated in an exploratory manner the relationships amongst different aspects of psychological inflexibility, emotional intelligence, and mental health symptoms (anxiety and depression) in school-aged children and adolescents. This was the first study exploring the experiential approach in children and adolescents. Mental health was predicted by avoidance and fusion of aversive private events and the experiential approach anxious clinging subscale. Depression was predicted by emotional intelligence. Results underline the bearing of psychological inflexibility for child/adolescent mental health. Likewise, the usefulness of emotional intelligence programs not only in clinical context but also in educative organizations, in order to reduce emotional disorders in these ages and contribute to a better psychological health in adulthood, must be unblemished.

In sum, the studies presented the relevance of personality in different contexts, as well as the importance of studying personality in designing interventions. Several conclusions are drawn from the results. First, self-esteem arises as a personality factor to be evaluated and improved before or in interaction with the mirror exposure therapy [1]. Second, individual differences on personality traits such as neuroticism and related factors such as intolerance to uncertainty and negative autofocus should be taken into consideration when designing prevention programs aiming to dampen the psychological impact of any sudden and unforeseen change in anyone’s life [2]. Third, and specially with respect to fibromyalgia, the patient’s individual personality and defense style evaluation is essential for a correct psychological diagnosis and treatment as well as to enhance mental health in fibromyalgia patients [7]. Fourth, the management of negative emotions related to high neuroticism are suggested to be essential for future intervention programs directed to migraine patients [6]. Fifth, early detection and regulation (by personality-targeted approach interventions and psychoeducation) of personality traits such as impulsivity or sensation seeking in young can prevent young patients from experiencing future traumatic events and therefore these patients from adolescent delinquency (e.g., sexual risk behaviors, substance abuse and misuse etc.). Additionally, early psychological intervention in children who have been exposed to trauma might hopefully lessen the risk to development of a hopelessness personality trait, which in turn is related to depression vulnerability [4]. Sixth, narcissistic traits in the general population may prevent from emotional/mental health concerns in some contexts [3]. Seventh, cluster C personality disorders have a similar impact in traumatization than cluster B personality disorders. In both cases, specialized screening and treatment programs are necessary and overall, those treating the preceding or concurrent trauma [8]. Last, psychological inflexibility in children and adolescent has the potential to predict future mental health problems. Hence, preventive interventions (e.g., based on Acceptance and Commitment Therapy) aimed at enhancing psychological flexibility in healthy children and adolescent might be of benefit handling aversive private events in this population and contribute to a better psychological health and general adjustment in adulthood [5].

Altogether, these studies underscore the importance of studying personality to prevent mental diseases and improve the effectiveness of intervention. Note that despite the findings presented in this Special Issue, the personality field still requires of continuous research in order to provide a more personalized and patient-centered approach, which benefit not only patients and their relatives but also the health system and society in general. Furthermore, other personality factors not explored in this special, but also prevalent in the youth population and associated with urgent health and social concerns, have to be studied (e.g., emotional dependence, social-network misuse, etc.).
